# Carriers in Cell-Based Therapies for Neurological Disorders

**DOI:** 10.3390/ijms150610669

**Published:** 2014-06-13

**Authors:** Francisca S. Y. Wong, Barbara P. Chan, Amy C. Y. Lo

**Affiliations:** 1 Department of Ophthalmology, Li Ka Shing Faculty of Medicine, The University of Hong Kong, Hong Kong, China; E-Mail: frannwong@gmail.com; 2 Department of Mechanical Engineering, Faculty of Engineering, The University of Hong Kong, Hong Kong, China; E-Mail: bpchan@hku.hk; 3 Research Center of Heart, Brain, Hormone and Healthy Aging, Li Ka Shing Faculty of Medicine, The University of Hong Kong, Hong Kong, China

**Keywords:** age-related neurodegeneration, drug delivery and drug-likeness, blood-brain barrier, biomaterials, cell encapsulation, microcarriers, central nervous system injury, peripheral nervous system

## Abstract

There is a pressing need for long-term neuroprotective and neuroregenerative therapies to promote full function recovery of injuries in the human nervous system resulting from trauma, stroke or degenerative diseases. Although cell-based therapies are promising in supporting repair and regeneration, direct introduction to the injury site is plagued by problems such as low transplanted cell survival rate, limited graft integration, immunorejection, and tumor formation. Neural tissue engineering offers an integrative and multifaceted approach to tackle these complex neurological disorders. Synergistic therapeutic effects can be obtained from combining customized biomaterial scaffolds with cell-based therapies. Current scaffold-facilitated cell transplantation strategies aim to achieve structural and functional rescue via offering a three-dimensional permissive and instructive environment for sustainable neuroactive factor production for prolonged periods and/or cell replacement at the target site. In this review, we intend to highlight important considerations in biomaterial selection and to review major biodegradable or non-biodegradable scaffolds used for cell transplantation to the central and peripheral nervous system in preclinical and clinical trials. Expanded knowledge in biomaterial properties and their prolonged interaction with transplanted and host cells have greatly expanded the possibilities for designing suitable carrier systems and the potential of cell therapies in the nervous system.

## 1. Introduction

Neurodegeneration resulting from trauma, stroke, or neurological diseases causes persistent and progressive loss of neuronal subtype, leading to permanent structural damage and functional impairment. Neurological disorders such as Alzheimer’s disease (AD), Parkinson’s disease (PD), Huntington’s disease (HD), amyotrophic lateral sclerosis (ALS), stroke, traumatic brain injury (TBI), spinal cord injury (SCI), retinal degeneration, and peripheral nerve trauma are amongst the most debilitating conditions of the current century. They impact on the entire age spectrum worldwide, causing prolonged reduction in the quality of life and creating heavy psychological and financial burden.

Scaffold-facilitated cell-based therapies are currently active areas of research in treating neural injuries resulting from trauma or disease. This review seeks to discuss the current state of the development of biomaterials served as cell-carrying scaffolds. Firstly, current challenges and emerging therapeutic strategies in repairing and regenerating the Peripheral Nervous System (PNS) and the Central Nervous System (CNS) are described, followed by the discussion of different approaches adopted in cell-based therapies. Then, the requirements for selecting desirable scaffolds are set out. Furthermore, present development states and applications of various scaffolding biomaterials are discussed. Lastly, conclusions and perspectives are presented for future work in developing scaffolds for treating neural disorders.

### 1.1. Current Challenges

In contrary to previous beliefs, neurons in both the PNS and the CNS demonstrate intrinsic abilities to regenerate under growth-supporting conditions. Aguayo and colleagues showed that CNS retinal neurons are able to regenerate within peripheral nerve grafts, but not beyond the graft into the CNS tissue [1]. However, due to the dissimilar post-injury physiological responses and associated glial cell functions, the local injury environment is permissive in the PNS yet inhibitory in the CNS. In the presence of nutrients, orientation, neurotrophic factors and myelin-digesting support provided by Schwann cells (SCs), regeneration of PNS neurons can take place spontaneously across a small nerve defect or be facilitated by direct end-to-end surgical reconnection. For larger nerve defects, the clinical golden standard, autologous nerve graft, is applied. However, nerve autografts are plagued by complications such as neuroma, donor site morbidity, nerve site mismatch and limited sources of donor tissue [2]. Complete functional recovery can rarely be achieved. Although axonal regeneration occurs, functional recovery may not always be the ultimate clinical outcome due to misdirection of regenerating axons towards an inappropriate target [3]. Therefore, there is interest in developing grafts with better functionalities as alternatives to autografts.

Nerve regeneration in the CNS is even more challenging. In response to the direct damage to the spinal cord, a cascade of secondary injury events that further expands the zone of cell death and tissue damage occurs. This leads to cell death via apoptosis and the establishment of barriers to regeneration. Destructive inflammation activates astrocytes and microglia, glial cells of the CNS, to proliferate and form glial scar in order to minimize further damage around the zone of necrotic cell death and protect uninjured tissue. The glial scar contains axonal growth inhibitory factors such as chondroitin sulfate proteoglycan and myelin-associated glycoproteins that limits axonal regeneration [4]. Most current therapies for the brain and the spinal cord are designed to pharmacologically modify disease symptoms but do not promote neural tissue repair or restoration of severed axonal connection. Trophic factors, anti-apoptotic drugs or anti-inflammatory drugs have been administered to limit the extent of secondary injury and to enhance the plasticity of undamaged circuits [5,6,7], however, their access to lesions in the CNS is often limited by numerous protective barriers surrounding the CNS, including the boney structures as well as the blood-brain-barrier and the blood-spinal cord-barrier [8]. Although drug administration via direct local injection can bypass the barriers, it leads to tissue damage. As for drugs that are able to cross the vascular barriers, the high doses required for systemic delivery can result in undesirable side effects. No therapies are yet available for full restoration of lost function over critical-sized nerve gaps or ongoing neurodegeneration in the PNS and the CNS [5,9].

### 1.2. Emerging Tissue-Engineering Treatment Strategies

In the past decade, several non-cell carrying polymeric nerve guidance conduits (NGCs) have been approved by the USA Food and Drug Administration (FDA) and Conformit Europe (CE) for bridging nerve gaps and promoting peripheral neural regeneration. The current development, design considerations and clinical outcomes of the approved NGCs were reviewed in [2,10]. NGCs simulate the natural regeneration process in the PNS by providing a permissive environment for neuroregeneration, mainly through physical guidance cues, and have obtained clinical success for PNS treatment [4]. When applied to the spinal cord, NGCs increase neural sprouting as well as reduce glial scar formation and the invasion of immune cells [11]. Interestingly, these results are commonly observed in scaffolds with varying compositions and architectural features. However, therapeutic outcomes of scaffolds with biomaterials alone are still less desirable than autographs for long lesions.

Tissue engineering integrates cell therapies and scaffolding technologies to replace damaged neurons and to reestablish the coaxial connection of neuronal circuits for functional recovery. With 3-D scaffolds, cell survival and graft integration can be augmented in cell transplantations. Scaffolds also prevent the random growth of regenerating axons by providing topographical guidance. They have demonstrated great potential to be translated into clinical practice. The next generation of tissue-engineered scaffolds for clinical application is likely to be engineered with: (1) mechanical and topographical cues; (2) biochemical cues such as neurotrophic factors and extracellular matrix (ECM) proteins; and (3) cells to guide and further enhance axonal regeneration [2,12]. Cell-based therapies are particularly warranted in cases where the complexities of injury or disease require multifaceted regeneration strategies. Recent combined therapeutic approaches incorporating factors such as cells, scaffolds and biochemical cues applied to the CNS were reviewed in [5].

Two main strategies of scaffold-facilitated cell transplantation are: (1) to create a permissive environment for regeneration by sustained protein delivery; and (2) to replace cell or tissue loss and promote functional repair. One strategy to replace the functional loss due to cell or tissue damage is to deliver polymer-encapsulated cells that are genetically programmed *ex vivo* for prolonged secretion of neuroactive factors. The supplement of supportive ECM components, neurotrophic factors, cell adhesion molecules, or anti-inflammatory cytokines promotes local cell survival and regeneration while attenuating secondary injury events. Cell encapsulation is particularly useful to deliver therapeutics that required local and controlled administration, such as neurotrophic factors with short half-lives and difficulties in systemic administration due to adverse side effects [13]. Cells of autogeneic, allogeneic and xenogeneic sources can be immunoisolated in macro- or microencapsulating devices with semipermeable membrane or matrix for prolonged and sustainable factor delivery *in vivo*. Current development and design considerations of cell encapsulation technology in the nervous system were reviewed in [13,14,15]. Another strategy is to seed or attach scaffolds with cells to replace supportive cells, neurons and other associated tissues. Cell choices, challenges and considerations for nerve tissue engineering [16], specifically for the brain [17], spinal cord [18] and PNS [12] were extensively reviewed. Most popular cell candidates include glial cells and stem/stem-like cells. Glial cells function to secrete neuroprotective agents that stimulate natural neural repair, whereas stem/stem-like cells have the additional potential to replace lost tissue via differentiating into neuronal cells. Modifying these cells to secrete neuroactive agents has further expanded their potential to aid repair in the nervous system [6]. Review of various types of adult cells used in neuroregeneration can be found in [17]. These scaffolds can take the form of hydrogel, macro- and microsized solid or particulate implants as well as pharmacologically active microcarriers (PAM) (review on PAM can be found in [19]). To date, numerous preclinical and clinical studies on scaffold-facilitated therapies for neurological disorders have demonstrated safety and efficacy.

Through tailoring biomaterial scaffolds to suit different cell transplantation approaches and intended periods of application, neuroprotection and neuroregeneration efficacies of cell therapies can be improved.

## 2. Criteria for Ideal Cell-Carriers in the Nervous System

Biomaterial-based delivery systems are essential in augmenting the therapeutic outcomes of cell therapies in neural tissue engineering. Ranging from hydrogel to macro- and microsized solid implants, there exist many options for conveying cells into the CNS and the PNS. Different tissue and neurological disorders require different biomaterial designs to achieve maximal neuroprotective and neuroregenerative outcomes (reviewed in [12,20,21]). In general, some of the most important considerations for choosing appropriate biomaterials include biocompatibility and biodegradability, biomechanical competency as well as functionalization with instructive cues to guide neuroregeneration.

### 2.1. Biocompatibility

Selection of biocompatible biomaterials for cell-carriers is important. Biocompatibility refers to the ability of a biomaterial to perform and support appropriate cellular behaviors without eliciting undesirable responses in the host, either locally or systemically, in specific applications (cited in [19]). When brought in contact with host tissue, biodegradable materials and their by-products should not induce hemolysis and damage to other blood components, coagulation and thrombus formation, toxic side effects to the surroundings, tumors, foreign body reactions and immune rejections [12].

### 2.2. Biodegradability

Biodegradability refers to an ability of a biomaterial to degrade controllably when implanted. Whether a biodegradable or non-biodegradable system should be used depends on the problem being faced and intended application. If immunoisolation is required, as in drug delivery applications, non-biodegradable or slow degrading systems over the intended period of implantation are desired. Their scaffolds serve as physical barriers to protect the cells from the host immune system and cell or tissue ingrowth while allowing sustained delivery of therapeutic proteins. An additional benefit is their ease of retrieval. Some clinical trials reported that non-biodegradable cell-encapsulating devices retrieved at treatment endpoints contained viable cells inside the membrane while no cells or tissues were found on their outer surfaces even though the patients received no immunosuppressant [22,23].

On the other hand, if the delivered cells are expected to differentiate into cells at the site and replace lost or injured tissues and functions, biodegradable systems are necessary. In these applications, non-degradable systems may be unfavorable since they require secondary surgeries for removal upon the maturation of the regenerated nerve and are prone to long-term complications such as compression syndrome [2]. For systems designed to degrade with time, it is important to ensure that the scaffold and its by-products are well tolerated by the transplanted cells and host, and can be eliminated [17]. The rate of biodegradation should be tuned to match the rate of nerve regeneration [12]. For systems that degrade too rapidly, neuroregeneration may be hampered by inadequate protection of transplanted cells from invading fibrous tissue or insufficient time for nerve fibers to mature [24]. Also, they will lead to an elevated local concentration of degraded by-products, which may be detrimental. For instance, accumulation of acidic by-products of polyesters due to mismatch in hydrolysis and metabolized rate poses harm to the transplanted cells and host tissue. However, if the degradation rate is too slow, chronic compression, foreign body reactions and mild inflammatory reactions may occur and require secondary surgeries for removal [2,12]. Thus, it is important to optimize biodegradation rates of cell delivery platforms to fulfill requirements of target organs and neurological condition as well as duration and therapeutic goals of the intervention.

When evaluating biodegradability, all factors contributing to scaffold degradation in native microenvironment should be considered. Instead of conducting simple *in vitro* degradability tests in water or phosphate buffered saline (PBS) at room temperature, assessment systems should simulate native degradation factors such as temperature, pH, ionic strength, hydrolysis and hence swelling, presence of enzymes and engulfing cells *etc*.

### 2.3. Biomechanical Competency

Biomechanical properties influence the performance of cell-delivery systems and subsequent neuroregeneration in the human body. Mechanical compliance affects cell behavior, fate and functioning [2]. During nerve regeneration in the PNS and the CNS, the cell-delivery systems should have nerve tissue matching mechanical strength to resist physiological loads and mechanical stresses from neighboring tissues without collapsing or losing their shape [12]. One way to obtain desirable mechanical properties is by combining polymers to integrate the merits of their constituents. For instance, blending poly(lactic-*co*-glycolic acid) (PLGA) with poly(l-lactic acid) (PLLA) can produce scaffolds that are more flexible but can still resist extensive elongation [25]. Also, wall thickness of NGCs has been identified in some studies as a contributing cause of neuroma formation [2]. Thinner walls should be used to ameliorate the problem. To ensure safety in application as well as survival and proper functioning of the implants, mechanical properties of the scaffolds should be carefully designed and evaluated.

Additionally, porosity and permeability are important mechanical considerations for cell-carrier design. Porous scaffolds or ones that become porous after gradual biodegradation *in* *vivo* allow host cell colonization, tissue ingrowth and vascularization [26]. These ingrowths help to minimize the mechanical irritation caused by relative shearing motion between the implant and host tissue [19,24]. Hence, necrosis and inflammation can be reduced and implants can perform with lower foreign body response. Moreover, vascularization of the systems allows better survival of transplanted cells and regenerated tissue, especially for larger-sized implants. For example, collagen guidance channels matrix with SC were vascularized in about a week and demonstrated the ability to support cell growth and proliferation after implantation [24]. Surface pores can promote host cell ingrowth at controlled degrees of cell spreading while allowing vasculature formation in close proximity to the host-material interface [19].

As for cell-encapsulating interventions, selective permeability of membranes or matrix is important in enabling the exchange of nutrient, oxygen, growth factors and metabolic products while preventing invasions of the host immune system. Long-term survival of the encapsulated cells and sustained factor delivery will be hindered if there are restrictions on the diffusive exchange of metabolic materials due to local reactions, such as the formation of a fibrous capsule on the implant [27]. Therefore, it is important to ensure that the biocompatibility, membrane thickness and properties as well as micro-architecture of these cell-delivery platforms are suitable for prolonged functioning [24]. Also, permeability of membrane should be designed to fit the specific metabolic requirements, such as oxygen and nutrients, of individual cell types. This is crucial especially during the initial phase of implantation when neovascularization is yet to occur [27].

### 2.4. Functionalization by Cues

Neural cells are highly responsive to physical, biochemical and electrical cues present in their microenvironment during growth, development and regeneration stages. Functionalization of biomaterials can artificially recreate the spatial and temporal presentation of these cues. This modifies the functions of scaffolds in promoting cell viability, directing cellular activities and instructing the neuroregeneration process.

Besides axonal outgrowth, the reestablishment of axonal connections is the key to successful functional recovery. Improved directional guidance can be achieved by incorporating physical cues in the scaffold design, which include bio-mimicking topographic cues and mechanical cues. Topographical cues include tailored surface patterning, such as nano- and microsized aligned fibers, grooves and pillars, as well as construct geometry, size *etc*. [28,29,30,31,32,33], (reviewed in [34]). Electrospinning [31,35,36,37,38], tethering of self-aligned polymeric scaffolds [39,40], photopolymerization [32], photocuring [33], and multi-channeled construction [41,42,43,44] are some of the promising ways to apply aligned features to scaffolds. Common 3-D scaffold processing techniques were reviewed in [45]. Mechanical properties such as scaffold stiffness [33,46], elasticity [47], pore structure [43,48,49], and tension [50,51] are also important considerations in cell-carrier design. Many studies demonstrated the effects of physical cues on regulating cellular attachment, alignment, migration, growth, gene expression, and differentiation lineage as well as limiting the random growth of regenerated axons [7,17]*.* Some *in vitro* studies reported that a higher fraction of neural stem/progenitor cells was committed to neuronal lineage when co-stimulated by biochemical differentiation cues and scaffolds with axially aligned features [52,53]. NGCs with micro-patterned inner lumens were able to influence transplanted cell alignment and neuroregeneration across sciatic nerve defects [28,29]. Aligned scaffolds enhanced neuronal alignments [31] and SC alignments [32,36,37,54] as well as axonal regeneration [39,40].

As for biochemical cues, surface modifications with neuroactive factors, ECM molecules, adhesive peptides, or other specific chemical structures that closely mimic endogenous materials are employed to enhance cell-material interaction [9,55]. For example, immobilizing the evolutionarily preserved cell-binding motifs Arg-Gly-Asp (RGD) or Ile-Lys-Val-Ala-Val (IKVAV) can improve cell-adhesion properties of the scaffold. These surface-bound biomolecules function similarly as physical topographic cues in modulating cell behavior, enhancing axonal regeneration and promoting more seamless device integration with the host tissue. Also, immobilized neurotrophic factors and drugs can be used to prolong drug delivery [56,57,58]. However, it is important to ensure that the immobilization process does not affect the efficacy and bioactivity of the drug [21]. Wang *et al.* showed that glial-cell derived neurotrophic factor (GDNF) protein covalently tethered onto electrospun nanofibrous scaffolds remained stable and promoted neural stem cell (NSC) survival and differentiation [56]. Implantation of the GDNF immobilized scaffold into rat brains showed an enhanced level of transplanted neural stem/progenitor cells survival, proliferation, migration, and neurite growth [57].

Another approach to further enhance the robustness of cell-carrying scaffolds is to incorporate soluble biochemical cues. As with other cues, these diffusible signals function to influence the viability, differentiation and behavior of the delivered cells. Also, the factors delivered may modulate the host environment by encouraging and accelerating the process of axonal regeneration, neutralizing local inhibitory signals, reducing inflammation, slowing scar formation and achieving better local cell infiltration and implant integration. Examples of common agents delivered are growth factors, cytokines and drugs such as anti-inflammatory dexamethasone and α-melanocyte stimulating hormone, or agents that prevent scar formation such as chondroitinase ABC (chABC) and myelin-associated inhibition factors (reviewed in [21]). Some studies encapsulate growth factors in biodegradable microspheres for sustained release while modifying their surfaces with ECM components for conveying cells to the brain [59,60,61,62,63,64,65]. In another study, chABC and cells were delivered in a non-biodegradable NGC to reduce inhibitory cues of scar tissue. The conduit was able to provide an environment for cell regeneration, enabled regenerated axons to leave the implant and improved the morphological and functional recovery in rats with SCI [66]. For soluble biochemical cues, the most common way of drug delivery is through mixing target drug with scaffold precursors during manufacturing processes, followed by scaffold degradation and drug diffusion after implantation. To achieve prolonged release, drugs are sometimes encapsulated within cross-linked hydrogels, microparticles, micro- or nanofibers, and even firstly entrapped in microspheres and then into other scaffolding materials [67]. The drug delivery rate and profile can be controlled by manipulating the physical properties of the biomaterials, such as pore size or cross-linking density and the design of the system.

Also, these adhesion molecules and soluble factors can be incorporated into biomaterial scaffolds in an isotropic or anisotropic manner, meaning that they are either uniformly distributed or directionally oriented. Although most biochemical cues have been presented uniformly *in vivo*, studies showed that axonal regeneration via anisotropic presentation of therapeutics was more effective than isotropic arrangements [68]. When compared with isotropic concentrations, biomolecules in gradients promote better growth cone chemotaxis and are more effective in directing axonal regeneration towards the distal direction [2,68]. Dodla *et al.* showed that NGCs with gradients of nerve growth factor (NGF) and laminin were able to promote neuroregeneration and functional rescue better than isotropic controls [69]. These anisotropic NGCs were reported to be able to match the performance of autograft over large peripheral nerve defects of 20 mm in rats. Other parameters to consider when designing anisotropic scaffolds include steepness of gradient, range of drug concentrations and drug candidates suitable for individual nerve types and sites of injury [67].

Electrical stimulation can be used to influence cellular growth and differentiation for neuroregenerative purposes. In one study, choroid plexus (CP) cells in combination with chronic electrical stimulation therapy resulted in synergistic neuroprotective effects in auditory neuron rescue [70]. To support neuroregeneration, an ideal neural scaffold should be electrical conductive. Electroactive conducting polymers such as polypyrrole, polyaniline and carbon nanotubes can support cell survival, proliferation, differentiation as well as neurite extension, axonal regeneration and functional recovery [6,12,71,72] (reviewed in [73]). Other materials used to prepare neural scaffolds include poly(phosphoesters), polyurethanes, and piezoelectric polymers [3]. In some studies, physical and biochemical cues are applied to these scaffolds to improve the cell-material interactions and biocompatibility [38,74,75].

Incorporation of physical, biochemical and electrical cues in cell-carrying biomaterials enables researchers to fabricate interactive and instructive cell-carriers that fit desired therapeutic targets and strategies.

## 3. Cell-Carriers with Non-Biodegradable Materials

Common non-biodegradable cell-carriers in neural tissue engineering include silicone, polyvinyl alcohol (PVA) and copolymer poly(acrylonitrile-*co*-vinyl chloride) (P(AN/VC)), polysulphone (PSU) and poly(ethersulphone) (PES), poly(ethylene terephthalate) (PET) and polypropylene (PP) (Table 1).

Although they are able to demonstrate stability over prolonged *in vivo* applications, their regeneration-inducing ability has been concluded to be inadequate when used alone [76]. In order to enhance their neuroregenerative properties, attempts have been made to incorporate cell supporting scaffolds and cells that are genetically modified and/or intrinsically able to secrete growth factors and therapeutic agents. Currently, the main applications of non-biodegradable materials include guidance channels as well as retrievable and semipermeable macroencapsulation devices for CNS applications.

### 3.1. Silicone

Since 1960s, silicone has been used as an inert and impermeable NGC to facilitate nerve regeneration following peripheral nerve and SCI. Silicone NGCs have provided an isolated environment impermeable to large molecules and ingrowth of scar tissue-forming cells while holding open a space through which regenerating cells and axons could grow [7,77]. However, early studies with empty or saline-filled conduits have only resulted in minimal nerve repair over distances up to 10 mm [3]. Also, they were plagued by chronic foreign body reaction that led the formation of fibrous scar tissue around the implants. Currently, silicone is mainly utilized as an experimental model for tubulization in peripheral nerve repair studies.

In order to induce neuroregeneration across large defects using silicone NGCs, various studies have incorporated cells into the conduits and functionalized the NGCs with natural biomaterials [76,78,79,80] (Table 1A). Neural progenitor cells (NPCs) [76], bone marrow mesenchymal stem cells (MSCs) [79,80] as well as SCs with or without modified neurotrophic factor expressions [78,81,82,83] have been applied to silicone conduits and transplanted across sciatic nerve defects in rats. Morphological regeneration and recovery in sensory and motor functions were observed in most cases when compared to control groups without cells. Also, multipotency of NPCs was maintained in these NGCs [76]. However, the issue of compression syndrome, which required secondary surgeries for removal, is yet to be addressed [2,84]. As a result, the focus of cell-carrying NGCs for spinal cord and peripheral nerve applications has been shifted to semipermeable or biodegradable materials.

### 3.2. Polyvinyl Alcohol (PVA) and Poly(acrylonitrile-co-vinyl chloride) (P(AN/VC))

Currently, the only commercially available non-biodegradable NGC scaffolds with FDA and/or CE approval is composed of PVA, named Salubridge™ and SaluTunnel™ from SaluMedica™ L.L.C. (Atlanta, USA). However, there has been no information about the repair and regeneration performance of the devices reported in peer-reviewed journals [2,10].

Retrievable implants with GDNF-expressing cells in PVA matrix with PES membrane, were applied in rat and non-human primate PD models and promoted neuroregeneration [85,86] (Table 1B). No major deleterious effects or inflammatory responses were induced by the multiple implantations of capsules into the same intraventricular/ periventricular space in attempt to increase the amount of GDNF delivered [86].

The most commonly applied co-polymer of PVA for cell encapsulation in neural tissue engineering is P(AN/VC) (Table 1C). Cell-encapsulating devices with semipermeable P(AN/VC) membrane has been used in AD, HD, PD and chronic pain studies. A range of cells, including genetically modified fibroblasts that expressed neurotrophic factors like NGF and ciliary neurotrophic factor (CNTF) [87,88,89,90,91,92] and dopaminergic cells such as pheochromocytoma (PC12) cells [93] and chromaffin cells [23,94] have been encapsulated. These cells were often suspended in a hydrogel matrix of collagen, alginate or Matrigel^®^. Prolonged implantation of these devices attenuated the neural damage induced and promoted behavioral rescue in rats and non-human primates of different neurological disorders. One study showed that when a P(AN/VC) macroencapsulated implant with PC12 cells was retrieved after extended implantation, functional levels of Parkinsonian rats returned to that of pre-implantation, demonstrating the therapeutic effects of these cells [93]. P(AN/VC) devices have showed to be well tolerated *in vivo* without showing adverse effects associated with direct systemic administration of neurotrophic factors. Upon retrieval, devices with asymmetric hollow fibers of P(AN/VC) and cell-seeded collagen matrix revealed numerous viable and mitotically active fibroblasts evenly distributed at high densities [87,88,92]. However, there was a general decrease in neurotrophic factor levels after prolonged implantation when compared with pre-implantation levels in these studies. In a Phase I clinical trial of chronic pain, a P(AN/VC)-coated alginate implant with xenogeneic chromaffin cells was applied to seven patients for about 1.5 to 6 months [23]. Although some improvements in pain rating and reductions in morphine intake were reported, the study did not control for placebo effects. Nevertheless, the device was retrievable and no host cells or fibrous coating on the surface of the implant in the absence of immunosuppressant was detected. In another study with P(AN/VC) conduits, combined drug and cell therapy of chABC, SCs and olfactory ensheathing glial cells showed their additive effects on and locomotory improvements in a rat SCI model [66].

### 3.3. Polysulphone (PSU) and Poly(ethersulphone) (PES)

Polysulfones are biocompatible and stable materials applied in cell encapsulation for CNS neural tissue engineering (Table 1D). Permeable PSU hollow fiber-based membranes have been used for encapsulating genetically modified fibroblasts that expressed neurotrophic factors, such as GDNF and brain-derived neurotrophic factor (BDNF). These implants showed neuroprotective and functional recovery effects in rat models of PD [95,96], stroke [97] and epilepsy [98]. Continuous neurotrophic factor delivery of the device was achievable for at least six months *in vivo* [96]*.* Although a few microglias and white blood cells were reported around the capsules, the immunoreactions were negligible and the devices were well tolerated [96,97].

A more commonly studied member of the family is PES (Table 1E). Cell-encapsulating PES devices have been applied in form of a hollow fiber-based membrane with cells delivered in empty [99], collagen-filled [22,100,101,102], PVA-filled [85,86] or PET yarn-filled [103,104,105,106,107] matrix. PES scaffold of the simplest macroencapsulating configuration has been applied to the hippocampus of a rat model of epilepsy [99]. A GDNF-expressing human cell line was encapsulated in a semipermeable PES membrane and achieved suppression in recurrent generalized seizures. Despite poor encapsulated cell survival in some of the devices, such cell death and subsequent lowered GDNF levels did not result in further host cell death and inflammation when compared with control groups. Death of encapsulated cells may explain the various degrees of glial scar formed around the implant, restricting material exchange of the entrapped cells with the host environment.

On the other hand, scaffolds with collagen fillers have been applied in various preclinical and clinical CNS neural disorder models. Genetically modified myoblasts that expressed CNTF were able to survive and remained functional after 3 months of implantation in rats with facial nerve axotomy [100]. Despite prolonged implantation in oxygen and nutrient straining environment, no accumulation of cell debris was observed in the retrieved implants. In a Phase I clinical study on ALS, a 5-cm-long implant of CNTF-expressing xenogeneic fibroblasts was introduced in the spinal cord of 6 ALS patients intrathecally with no immunosuppressant [22]. After 17 weeks of implantation, the extracted device remained intact with viable cells encapsulated. The outer surface was devoid of tissue and cell adhesion. In another 2-year Phase I clinical study, a similar device of 2.5-cm-long was implanted into 6 patients with HD [101,102]. Improvement in electrophysiological results was correlated with the amount of CNTF secreted from the implant. Although the retrievable implant remained intact and was well tolerated after prolonged application *in vivo*, the frequent replacement of the device every 6 months limited its practical use for chronic neurodegenerative diseases. Also, variations in *in vivo* CNTF levels and cell viability between the patients were reported in these studies. The authors speculated that fibroblast cell death was due to reduction of the inner volume of the implant. The reasons for such reduction were the occurrence of cell death and the presence of necrotic debris under oxygen- and nutrient-strained conditions with continuous cell proliferation. Moreover, the difference in cell number may be attributed to differential residence time *in vitro* [22]. These studies demonstrated the importance of cell selection for practical application.

Another design of PES implants involving a PET internal scaffold has been applied to tackle chronic retinal disease in various preclinical and clinical trials. The safety, drug release profile and efficacy has been studied in rat and canine models of retinal degeneration [103], a rabbit model [104] as well as human patients with retinitis pigmentosa (RP) [105,106,107], geographic atrophy (GA) associated with dry age-related macular degeneration [108] and Usher syndrome type 2 [106]. In these studies, CNTF-secreting human retinal pigment epithelium cells (RPE cells) were seeded in a scaffold of PET yarn and macroencapsulated by PES membrane. As the preclinical studies demonstrated a continuous production CNTF output of up to 1 year at therapeutic levels [104] and neuroprotective results in various animal models [103], a 6-month Phase I clinical study of the CNTF-producing Renexus^®^ (NT-501) device (Neurotech Pharmaceuticals, Inc., Lincoln, NE, USA) was carried out on 10 patients with advanced RP [105]. The implants were well tolerated with no severe ocular inflammation detected, despite one reported case of surgically-induced choroidal detachment. Cell populations in the implant at the end of the study remained healthy.

After demonstrating the safety and feasibility of NT-501 for extended implantations, two follow-up studies supported by Neurotech Pharmaceuticals, Inc. were carried out. The first was a 12-month Phase II trial involving 51 patients with GA [108], and the second was a Phase II longitudinal study of 30 to 35 months recruiting two patients with RP and one with Usher syndrome type 2 [106]. NT-501 and the implantation process were well tolerated in both studies, with no reported treatment-related or systemic adverse effects over 12 and 24 months of study. In GA patients, NT-501 promoted structural improvements in terms of higher retinal thickness and macular volume. Improvements were detectable as soon as 4 months after implantation in higher CNTF dosage group and were maintained throughout the 12-month period. Such changes were associated with stable and maintained visual functions, measured by mean best-corrected visual acuity change. Yet, there were no changes in electroretinogram (ERG) responses and visual field sensitivity in the treated eyes compared with the baseline. As for patients with RP, structural rescue was shown in the thickness of outer retinal layers and lower cone density decrease in CNTF-treated eyes than in sham-treated eyes. The cone spacing resulting from CNTF treatment was comparable to normal eyes studied over similar periods of time. However, there were no significant changes in visual function in terms of visual acuity, visual field sensitivity, or ERG responses over 30 months for patients in the longitudinal study [106]. Such absence of changes may be due to the limitations imposed by the nature of disease progression, and sensitivity limitations of clinical functional evaluations employed for this population of patients. As a result, the patients were evaluated by a more sensitive technique called adaptive optics scanning laser ophthalmoscopy that targets individual cone photoreceptors. Results suggested that there might be a slowing down in the progression of retinal degeneration in these patients. Analysis on NT-501 explanted from patients demonstrated the stable performance of the implant. The device was able to maintain a stable cell number, cell viability and CNTF output over 2 years [107]. CNTF, anti-CNTF antibodies, and antibodies to the encapsulated cells were not detected in the serum of patients at all time points. Yet, larger sample size was warranted for both studies as well as longer study duration for evaluating the effect of CNTF-treatment on GA progression. Also, there was a need for higher-resolution imaging techniques and evaluations on more retinal locations to provide a more sensitive analysis on the effect of CNTF treatment in cone structures in the longitudinal study.

### 3.4. Polypropylene (PP)

PP is usually applied for macroencapsulation for CNS neural tissue engineering (Table 1F). A porous PP filter with encapsulated neurotrophic factor-expressing fibroblasts was applied to rodent models of facial nerve axotomy and progressive motor neuronopathy [109,110,111]. CNTF-producing implants yielded neuroprotective effects on motor neurons, functional rescue and increased survival time [110,111]. Although the implants were generally well tolerated, a small amount of fibrotic tissue growth around the implant was detected without cell penetration into the construct [110].

**Table 1:** Examples of non-biodegradable cell-carriers applied in preclinical animal studies and clinical trials.

Biomaterial	Form of Scaffold	Cell Type & Modified Factor Expression (if any)		Animal & Neurological Disorder Model	Outcomes	Ref.
**(A) Silicone**	Tubular NGC			PNS		
	(Hollow)	SC & SC-GDNF		Rat sciatic nerve defect (10 mm)	Improved axonal regeneration, nerve conduction velocity and compound muscle action potential.	[82]
	With rat plasma	SC		Rat sciatic nerve defect (10 mm)	Improved axonal regeneration.	[81]
	With Matrigel^®^	SC & SC-FGF-2 isoforms		Rat sciatic nerve defect (10 mm)	Improved axonal regeneration, sensory and motor recovery. Different isoforms of FGF-2 exerted different effects on the regenerating axons.	[78]
	With Pluronic F127 gel and PGA	SC		Nude rat sciatic nerve defect (10 mm)	Constricted and limited axonal regeneration with some SFI improvements.	[83]
	With collagen	NPC		Rat sciatic nerve defect (15 mm)	Improved axonal regeneration with detectable action potentials. Part of the transplanted cells differentiated into SC-like supportive cells.	[76]
	With gelatin	MSC		Rat sciatic nerve defect (15 mm)	Improved axonal regeneration, ameliorated loss of gastrocnemius muscle mass. Enhanced walking behavior and SFI recovery.	[79]
	With laminin-coated multi-walled chitosan insert	MSC		Rat sciatic nerve defect (10 mm)	Improved axonal regeneration, ameliorated loss of gastrocnemius muscle mass and enhanced SFI recovery.	[80]
**(B) PVA**	Porous cylindrical matrix filler of cell-encapsulating device			CNS		
	In PES membrane	Fibroblast–GDNF		Rat PD model (6-OHDA induced)	Improved movement initiation and swimming performance. After the withdrawal of device, behavioral and morphological improvements were maintained until the sacrifice.	[85]
		Myoblast-GDNF		Non-human primate PD model (MPTP-induced)	Increased volume of the cell bodies, exerted neuroprotective effects and achieved transient recovery of motor deficits.	[86]
**(C) P(AN/VC)**	Hollow fiber-based membrane of cell-encapsulating device			CNS		
	(Hollow)	Chromaffin cell		Rat chronic pain model	Supported transplanted cell survival. Repeatedly reduced pain sensitivity with nicotine stimulation.	[94]
	With alginate	Chromaffin cell		Phase I clinical trial: Chronic pain patients	Devices were retrievable. Supported transplanted cell survival without immunosuppressant. Yet, only moderate therapeutic outcomes, including morphine intake and pain ratings improvements, were detected in some patients.	[23]
	With Matrigel^®^	Fibroblast-NGF		Rat AD model (fimbria and dorsal fornix aspiration)	Ameliorated loss of septal choline acetyltransferase expression.	[92]
	With collagen	Fibroblast-NGF		i. Rat HD model (QA-induced); ii. Non-human primate AD model (fornix transection or aspiration)	Protected neurons from induced damages. Reduced rotational behavior in rodents.	[87,90,91]
		Fibroblast-CNTF		Rat and Non-human primate HD model (QA-induced)	Reduced the extent of induced striatal damage. Exerted trophic influence on degenerating striatal neurons as well as on critical non-striatal regions in non-human primates. Improved rotational behavior but not skilled forelimb function in rodents.	[88,89]
		PC12		Rat PD model (6-OHDA induced)	Continued presence of intrastriatal implants was required to maintain the reduction in rotation behavior. Device output was affected by the site of implantation.	[93]
	Tubular NGC			CNS		
	With Matrigel^®^ and release of chABC	SC and OEG		Rat SCI Model (transection)	Improved axonal regeneration and enhanced coupling of forelimb and hindlimb.	[66]
**(D) PSU**	Hollow fiber-based membrane of cell-encapsulating device			CNS		
	(Hollow)		Fibroblast-GDNF	i. Rat PD model (6-OHDA induced) ii. Rat stroke model (hypoxia-ischemia induced)	Ameliorated neuronal damage reduced infarct volume. Improved rotational behavior in PD model.	[95,96,97]
			Fibroblast-BDNF	Rat epilepsy model (kainic acid induced)	Exerted neuroprotective effects with enhanced neurogenesis, ameliorated seizure stage and number of abnormal spikes.	[98]
**(E) PES**	Hollow fiber-based membrane of cell-encapsulating device			CNS		
	(Hollow)		(human cell line)-GDNF	Rat epilepsy model (electrically induced)	Suppressed recurrent generalized seizures.	[99]
	With porous PVA matrix		Fibroblast-GDNF	Rat PD model (6-OHDA induced)	Improved movement initiation and swimming performance. After the withdrawal of device, behavioral and morphological improvements were maintained until the sacrifice.	[85]
			Myoblast-GDNF	Non-human primate PD model (MPTP-induced)	Increased volume of the cell bodies, exerted neuroprotective effects and achieved transient recovery of motor deficits.	[86]
	With collagen		Myoblast-CNTF	Rat ALS model (facial nerve axotomy)	Protected motor neurons from induced cell death.	[100]
			Fibroblast–CNTF	i. Phase I clinical trial: ALS patients; ii. Phase I clinical trial: HD patients	Prolonged device implantation and CNTF delivery were well tolerated. There were a varying number of encapsulated cells, hence, CNTF levels.	[22,101,102]
**(E) PES**	With PET yarn; Renexus^®^ (NT-501) device (Neurotech Pharmaceuticals, Inc.)		RPE cell-CNTF	i. Rat and canine retinal degeneration models (S334ter-3 and Rcd1 respectively); ii. Healthy rabbit; iii. Phase I clinical trial: RP patients; iv. Phase II clinical trial: GA patients; v. Phase II clinical trial: RP and Usher syndrome type 2 patients	Supported continuous factor production, transplanted cell survival and exerted neuroprotective results. Well tolerated in a Phase I clinical study. Promoted sustainable structural and some visual function improvements in Phase II trials.	[103,104,105,106,107,108]
**(F) PP**	Porous filter membrane			CNS		
	(Hollow)		Fibroblast-CNTF or GDNF	Mouse ALS model (pmn/pmn)	Exerted neuroprotective effects on motorneurones. CNTF treatment improved motor function recovery. GDNF treatment slowed motorneuron loss but not axonal degeneration or premature death of mice.	[109,111]
			Fibroblast-CNTF	Rat ALS model (facial nerve axotomy)	Exerted neuroprotective effects on motorneurones.	[110]

Abbreviations: AD: Alzheimer’s disease, ALS: amyotrophic lateral sclerosis, BDNF: brain-derived neurotrophic factor, MSC: bone marrow mesenchymal stem cell, chABC: chondroitinase ABC, CNS: central nervous system, CNTF: ciliary neurotrophic factor, FGF-2: fibroblast growth factor-2, GA: geographic atrophy associated with dry age-related macular degeneration, GDNF: glial cell-derived neurotrophic factor, HD: Huntington’s disease, MPTP: mitochondrial complex I inhibitor 1-methyl-4-phenyl-1,2,3,6-tetrahydropyridine, NGC: nerve guidance conduit, NGF: nerve growth factor, NPC: neuronal progenitor cell, OEG: olfactory ensheathing glia, P(AN/VC): poly(acrylonitrile-*co*-vinyl chloride), PC12: pheochromocytoma cell, PD: Parkinson’s disease, PES: poly(ethersulphone), PET: poly(ethylene terephthalate), PGA: poly(glycolic acid), pmn: progressive motorneuronopathy, PNS: peripheral nervous system, PP: polypropylene, PSU: polysulfones, PVA: polyvinyl alcohol, QA: quinolinic acid, RP: retinitis pigmentosa, RPE: retinal pigment epithelium cell, SC: Schwann cell, SCI: spinal cord injury, SFI: sciatic function index, 6-OHDA: 6-hydroxydopamine.

## 4. Cell-Carriers with Biodegradable Materials

Synthetic and natural biodegradable materials are very attractive candidates for transplanting cells into the human nervous system for prolonged local factor delivery and stimulating neuroregeneration and tissue re-organization. As they have generally good biocompatibility and can be tailored with desirable degradation rate, mechanical and biochemical properties, they are actively studied for neural tissue engineering. Commonly used synthetic biodegradable materials include polyesters (Table 2) and poly(ethylene glycol). As for natural polymers, ECM-based materials and their derivatives as well as other naturally occurring biopolymers are promising choices to facilitate cell therapies in the CNS and the PNS (Table 3).

### 4.1. Commercially Approved Biodegradable Nerve Conduits

Currently, the majority of FDA and/or CE approved nerve conduits/cuffs/wraps are biodegradable [10]. These include collagen-based NeuraGen^®^ and NeuraWrap™ (Integra Life Sciences Corp., Plainsboro, NJ, USA); NeuroMatrix™, Neuroflex™ and NeuroMend™ (Collagen Matrix, Inc., Franklin Lakes, NJ, USA); and RevolNerv^®^ (Orthomed S.A.S., Lyon, France) as well as synthetic polyester-based Neurotube^®^ nerve guidance (Synovis Micro Companies Alliance. Birmingham, AL, USA) and Neurolac^®^ nerve guidance (Polyganics B.V., Groningen, The Netherlands). Their degradation rates range from 3 months to 4 years.

Experimental and clinical results showed that these conduits were able to achieve comparable or even more superior neuroregenerative outcomes than autografts in relatively small nerve defects [2,3]. However, studies suggest that some of these devices were plagued by issues such as biocompatibility, swelling, degradation rate, automutilation, rigidity and patient complications [2]. Also, for critical-sized gaps, processed nerve allograft Avance^®^ allograft (AxoGen Inc., Alachua, FL, USA) may be a better choice. Up to date, limited information is available in peer-reviewed articles regarding NeuroMatrix™, Neuroflex™, NeuraMend™ and NeuraWrap™ [10]. The polymer constituents of these approved conduits are not functionalized with biochemical or physical cues or cells. Therefore, these conduits fulfill only the basic criteria of desirable nerve guides [10]. Cell incorporation should be one of the focuses when designing the next generation’s nerve implants.

### 4.2. Synthetic Biomaterials

Synthetic biomaterials are extensively explored for neural tissue engineering. Although they do not have the inherent bioactivity of natural materials, they are advantageous because of their tunable mechanical and biochemical properties. Their product uniformity and ease of scaling up for manufacturing and ease of modifying with biochemical, physical and electrical cues are some of the advantages over natural biomaterials [112]. Commonly used synthetic biodegradable cell-carriers are polyesters and poly(ethylene glycol) (PEG).

#### 4.2.1. Polyesters

Due to their well-established *in vivo* biocompatibility, safety and passing of regulatory approval, aliphatic polyester devices are widely used in clinical settings and neural tissue engineering. Polyesters are attractive materials as cell-carriers because they can be tailored with suitable surface chemistry for cell attachment, proliferation, and differentiation [113]. Poly(glycolic acid) (PGA), poly(lactic acid) (PLA) and their co-polymer poly(lactic-*co*-glycolic acid) (PLGA) are among the most used polyesters for cell delivery. The two FDA and CE approved nerve conduits, Neurotube^®^ and Neurolac^®^, are composed of PGA and poly-dl-lactide-*co*-ε-caprolactone, respectively. The effectiveness of polyester cell-carriers for CNS and PNS applications have been extensively evaluated in many preclinical trials as illustrated in Table 2.

PLA and PGA are biodegraded via hydrolyzation into metabolites lactic acid and glycolic acid and are eliminated by natural metabolic pathways [114]. The biodegradation rate of polyesters depends on hydrophobicity and degree of crystallinity. PGA has a more hydrophilic and crystalline structure than PLA. Thus, the typical degradation rate of PGA is in weeks while PLA is over a year [114]. PLA exists in two chiral forms, poly(l-lactic acid) (PLLA) and poly(d-lactic acid) (PDLA). The faster degrading poly(d,l-lactic acid) (PDLLA) has a more amorphous structure than PLLA or PDLA. Depending on the requirements of individual applications, adjusting the copolymer ratio of lactic acid to glycolic acid of PLGA changes their biodegrading rates that range from less than a month to several years. Biodegradation that is too slow is undesirable because the encasing of regenerated nerve by foreign material over extended periods tends to develop compression syndromes and evoke foreign body reaction [41,115]. However, if the implant is degraded too fast, its by-products may cause pH changes around the implantation site and damage donor cells and host tissue. Therefore, the tunable degradation rate and mechanical properties of polyesters offers potentials in developing customized therapies for neural tissue engineering.

##### Poly(lactic acid) (PLA) and Poly(glycolic acid) (PGA)

As PGA has a relatively short biodegradation time, it has been explored as a temporary scaffolding material to facilitate donor cell functioning and ultimate cell replacement (Table 2A). PGA and its biodegraded by-products are well tolerated and do not create toxic inflammatory reactions [116]. SC containing PGA scaffolds were able to support neuroregeneration without the need of supportive guidance tubes in nude rats with 10 mm sciatic nerve defect after 2 months of implantation [83]. Park *et al.* showed that PGA fibers were able to support NSC survival, attachment and differentiation into various neuronal cell types in the brain [116]. When implanted into the cavity of mice with cerebral palsy, PGA scaffolds functioned as a template for the gross morphology of the regenerated parenchyma. Implantation of the construct resulted in the regeneration of a vascularized parenchyma with some reconstituted anatomical connections as well as functional improvements. This study suggested that PGA scaffolds were able to minimize the secondary injury response of the injured brain, which included inflammation and scarring.

Longer-lasting PLLA and PDLLA scaffolds have been studied as cell-seeded biodegradable nerve conduits (Table 2B,C). Although PLLA has good processability, its slow degradation rate may not be ideal for peripheral neuroregeneration [117]. On the other hand, cell-based PDLLA NGCs have demonstrated biocompatibility and neuroregenerative properties as conduits in the CNS and the PNS. Vascularization and modest structural and functional regenerations were observed 6 weeks after the implantation of a porous PDLLA scaffold with D15A-secreting SCs into rats with transected SCI [118]. The mild regeneration reported might be correlated with the substantial cell death occurring during the first days and the low transplanted SC survival rate *in vivo*. As a result, there might not be adequate levels of neurotrophic factors within the scaffold to yield significant therapeutic effects. In some studies, combined therapeutic approaches employing cells and mechanically functionalized PDLLA scaffolds have enhanced neuroregeneration in rodent models. A SC-seeded PDLLA NGC with micropatterned inner lumen was implanted into rats with 10 mm sciatic nerve gap [29]. These NGCs were able to enhance the rate of recovery and functional rescue despite having a similar number and size of regenerated axons with control groups (without SCs and/or without micropatterning). In a separate study, Hsu *et al.* implanted a micropatterned-PDLLA conduit seeded with NSCs to bridge a 10 mm sciatic nerve gap in rats [28]. Nerve structural regeneration and functional recovery were detected upon 6 weeks of implantation.

##### PLGA

PLGA is one of the most studied biomaterials for supporting cell transplantation therapies in the CNS and the PNS (Table 2D). Some of the currently pursued cell-based applications of PLGA include cell incorporated nerve conduits for the PNS and the spinal cord, as well as, microcarriers for the brain. They are often used in conjunction with SCs or stem/stem-like cells that are designated for cell replacement and growth factor delivery. 

To examine the effects of cell incorporation in PLGA scaffolds, Teng *et al.* implanted a multicomponent PLGA and PLGA-polylysine blending scaffold with NSCs into an adult rat hemisection SCI model [119]. The scaffold was constructed with a biodegradable, yet cell-ingrowth inhibitory, outer zone. The device was prepared to achieve a degradation rate of about 30 to 60 days. Scaffolds, either with or without cells, were able to reduce the lesion, prevent the formation of scar tissue and cysts as well as improve motion and sensory recovery. Although the scaffold alone can support neuroregeneration, such an effect was significantly augmented with the incorporation of cells. The study also suggested that NSC might function to provide trophic support rather than cell replacement as they were found undifferentiated in the spinal cord regardless of the presence of scaffolds.

Other studies involving cell-containing PLGA NGCs have demonstrated functional improvements in animal models of peripheral nerve injury to different extents. PLGA (85:15) foam scaffolds with SCs showed good *in vivo* tolerability and gradual degradation in rats with 10 mm sciatic nerve gap over 12 weeks of application [115]. Although cell-seeded PLGA conduits have a lower total axon count than non-seeded controls, the seeded implants with glial growth factor (GGF) pretreatment showed a higher degree of myelination on the regenerated axons and fastest conduction velocities. In another study, 20 mm sciatic nerve gap in rabbits was bridged by laminin-coated and SC-seeded PLGA (90:10) scaffolds [120]. 8 weeks after the implantation, comparable morphological rescues in mid-graft, including the number of axons and area of nerve fibers, were observed in the seeded implants and were comparable to autografts. Shi *et al.* further investigated the combined effect of cell replacement therapy with sustained neurotrophic factor delivery [121]. NSCs and SCs were modified to express GDNF and infused into PLGA tubes followed by implantation into rats with facial nerve transections in two separate sets of experiments. The conduits degraded within approximately two months after implantation. Nerve action potential amplitude, axonal area, and axonal number were significantly greater in both types of genetically modified cells when compared with unmodified NSCs.

Some studies have focused on incorporating guidance cues into cell-based PLGA scaffolds to enhance conduit performance. Topographical patterns on PLGA-based scaffolds have been shown to influence neuronal migration, SC orientation and their outgrowth [31,37]. Hadlock *et al.* implanted a five-channeled scaffold with laminin-coated PLGA (85:15) foam and SCs to a 7 mm sciatic nerve gap in rats [41]. After 6 weeks, regenerated fibers were detectable in each of the channels. When compared to autograft, similar amounts but larger axonal widths of regenerated neuronal tissue were identified in the mid-conduit region in the seeded-PLGA group. This multi-channeled configuration was able to increase the surface area for SC migration, adhesion and alignment while being pliable enough to be bent into gentle curves for bridging gaps. However, the study did not examine the functional rescue following implantation. In another study, chitosan conduits with aligned PLGA fiber inserts and MSCs showed better axonal regeneration and functional recovery than scaffolds only when implanted across a large sciatic nerve gap of 60 mm in dogs and a median nerve defect of 50 mm in non-human primate for 12 months [122,123]. Besides mechanical cues, neuroactive factors and natural materials such as collagen, laminin, fibronectin and chitosan are often applied to PLGA scaffolds as biochemical cues to promote cell survival, attachment, proliferation and differentiation [7,124,125,126].

Enhanced mechanical flexibility and resistance to extensive elongation can be achieved by blending PLGA with PLLA [25] (Table 2E). The faster-degrading PLGA functions to facilitate cell ingrowth while the slower-degrading PLLA provides mechanical stiffness to support the scaffold [124]. The PLGA-PLLA matrix has been shown to support cell survival, migration and differentiation in some studies. Levenberg *et al.* showed that a PLGA-PLLA scaffold was able to support neural lineage differentiation of human embryonic stem cells *in vitro.* When treated with NGF and neurotrophin-3 (NT-3), neural rosette-like structures as well as vascular structures were found throughout the scaffolds [63]. Also, in another study, a PLGA-PLLA scaffold with retinal progenitor cells showed much higher cell survival and overall cell yield in mice subretinal region when compared with direct injection of cells after 4 weeks [64]. Enhanced survival, differentiation as well as migration and integration of grafted cells with host retina were detected. The device was gradually degraded with increasing pore sizes.

Apart from nerve conduits, PLGA-based microspheres or PAMs have sparked lots of investigations as a combined therapeutic approach to convey cells while providing controlled drug delivery and temporary scaffolding that conforms to the transplanted site. The released growth factor can modify the host microenvironment as well as stimulate transplanted cell survival and direct the differentiation of grafted cells [65]. Several PAM systems for the brain have been developed over the past decade. Mahoney *et al.* proposed a cell delivery system with surface modified PLGA (50:50) microspheres for conveying embryonic neurons from the rat cortex to the brain [60]. When transplanted into rats, the cells differentiated into cholinergic neurons. Another NGF-releasing PAM system that conveyed PC12 cells, with PLGA (37.5:25) microspheres resulted in improvements in behavior tests in a PD rat model [59]. Following this success, GDNF-releasing PAMs with fetal ventral mesencephalon cells adhered on PLGA (37.5:25) microcarriers was developed [61]. These PAMs degraded significantly after 8 weeks *in vitro.* Rats implanted with these PAMs showed better cell survival, fiber outgrowth and behavioral response than controls with injection of cells alone. Also, a larger graft volume of PAM was detected. In a separate study by Bible *et al.*, PLGA (85:15) microspheres with attached NSCs were created [62]. *In vitro* studies showed that the attached cells retained their stem cell status, viability and ability to proliferate. Implantion of these particles into a stroke rat model without immunosuppression showed that they were able to fill out the lesion cavity and provide structural support for the transplanted cells. They integrated efficiently within host tissue forming a primitive neural tissue. Some cells migrated along other particles or into the surrounding tissue, while some remained on the PAM. Aiming to establish neovascularization within the PAM network, they have further implanted vascular endothelial growth factor-releasing PAM with immortalized NSCs into a rat stroke model [127]. Endothelial cells from the host were attracted into the primitive neural tissue and formed a neovasculature in some parts, whereas in other parts, these cells merely interspersed with the transplanted cells. Evidence of hypervascularization was detected. Another PLGA (37.5:25) carrier system was designed to deliver marrow-isolated adult multilineage-inducible cells (MIAMI cells), which are a homogeneous subpopulation of immature human mesenchymal stromal cells [128]. Epidermal growth factor and basic fibroblast growth factor pretreated MIAMI cells were able to protect Cornu Ammonis layer 1 neurons from ischemic death in *ex vivo* hippocampal slices and rats with global ischemia.

#### 4.2.2. Poly(ethylene glycol) (PEG)

PEG is another class of synthetic polymers investigated for facilitated cell delivery. PEG is useful in minimizing the immune response following implantation due to its resistance to protein adsorption and cell adhesion [21]. PEG-hydrogels with varying degradation rates can be achieved by different cross-linking methods. To improve cell infiltration and attachment properties, PEG can be blended with other biopolymers or modified with sites for cell adhesion.

*In vitro* studies showed that PEG was able to support neural precursor cell survival, proliferation and differentiation into different neural cell types [129]. Also, these cells were able to tolerate the ultraviolet light-induced PEG gelation process. To achieve a more uniform seeding and earlier neural process extensions, Namba *et al.* used fibrin (to be removed after PEG gelation) as a template for creating a network of interconnected pores [130]. With the presence of different biochemical cues, the neuronal compositions within the PEG hydrogel can be controlled [131]. Further *in vivo* investigations would be needed to further demonstrate its usefulness in neural tissue engineering.

**Table 2:** Examples of synthetic, biodegradable polyester cell-carriers used *in vivo* in preclinical studies.

Biomaterial		Form of Scaffold	Cell Type & Modified Factor Expression (if any)	Animal & Neurological Disorder Model	Outcomes	Ref.
**(A) PGA**		Scaffold NGC		PNS		
		With Pluronic F127 gel	SC	Nude rat sciatic nerve defect (10 mm)	Improved axonal regeneration. SFI recovery was comparable to autograft.	[83]
		Scaffold with small fibers in a woven array		CNS		
		-	NSC	Mouse cerebral palsy model (hypoxic-ischemia induced)	Scaffold filled lesion. Reduced parenchymal loss with regenerated meshwork of hostand donor-derived neurons and neo-vascularization. Diminished unilateral rotation.	[116]
**(B) PLLA**		Tubular NGC		PNS		
		With collagen	SC	Rat sciatic nerve defect (12 mm)	Limited axonal regeneration and SFI recovery.	[117]
**(C) PDLLA**		Tubular NGC		PNS/CNS		
		With micropatterned inner lumen (non-porous)	NSC	Rat sciatic nerve defect (10 mm)	Improved axonal regeneration, vascularization and SFI recovery.	[28]
		With micropatterned inner lumen (porous)	SC	Rat sciatic nerve defect (10 mm)	Improved axonal regeneration rate and SFI recovery.	[29]
		With fibrin	SC-D15A	Rat SCI Model (transection)	Increased vascularization but limited axonal regeneration response into and across the scaffold as well as hindlimb function recovery.	[118]
**(D) PLGA**		Tubular NGC		PNS		
		(Hollow)	NSC-GDNF & SC-GDNF	Rat facial nerve defect (transection)	Improved axonal regeneration and action potential recovery.	[121]
		With five channels & laminin coating (foam matrix)	SC	Rat sciatic nerve defect (7 mm)	Improved axonal regeneration comparable to autograft in mid-conduit region with axonal fibers detectable in all channels.	[41]
**(D) PLGA**		With GGF (foam matrix)	SC	Rat sciatic nerve defect (10 mm)	Improved axonal regeneration and conduction velocities.	[115]
		Of PLGA membrane and fiber matrix, with laminin and rat tail glue coating	SC	Rabbit sciatic nerve defect (20 mm)	Improved axonal regeneration at mid-graft comparable to autografts.	[120]
		Of chitosan conduit, with PLGA fiber inserts	MSC	Canine sciatic nerve defect (60 mm)	Improved axonal regeneration and functional recovery.	[122]
			MSC	Non-human primate median nerve defect (50 mm)	Improved axonal regeneration and functional recovery.	[123]
		Multicomponent Scaffold		CNS		
		PLGA and PLGA-polylysine blend, with two compartments	NSC	Rat SCI model (hemisection)	Improved axonal regeneration, reduced lesion size and glial scarring. Improved motion and sensory functions.	[119]
		PAM/ microcarrier		CNS		
		With NGF release and coating with polylysine	Embryonic neuron from rat cortex	Healthy SD Rat	Supported transplanted cell survival and differentiation.	[60]
		With NGF release and coating with polylysine and fibronectin-like synthetic molecules	PC12	Rat PD model (6-OHDA induced)	Supported transplanted cell differentiation and reduced cell death and proliferation. Reduced rotational behavior.	[59]
		With GDNF release and polylysine coating	FVM cell	Rat PD model (6-OHDA induced)	Improved transplanted cell survival, fiber outgrowth and reduced rotational behavior.	[61]
		With allylamine deposition and fibronectin coating	NSC	Rat stroke model (MCAO induced)	PAM filled lesion cavity. Scaffold integrated efficiently within host tissue forming a primitive neural tissue.	[62]
**(D) PLGA**		With VEGF release and laminin coating	Immortalized NSC	Rat stroke model (MCAO induced)	Attracted host endothelial cells into scaffold, established neovasculature in some parts and merely interspersed with NSCs in other parts. Evidence of hypervascularization present.	[127]
		With fibronectin and polylysine coating	MIAMI cell	Rat stroke model (global cerebral ischemia induced)	Protected neurons in Cornu Ammonis layer 1 hippocampal region from ischemic death.	[128]
**(E) PLGA-PLLA**		Scaffold		CNS		
		PLGA and PLLA blend, with laminin coating	RPC	Mouse rho^−/−^ model	Supported transplanted cell survival, differentiation, migration and integration into host retina.	[64]

Abbreviations: CNS: central nervous system, GDNF: glial cell-derived neurotrophic factor, GGF: glial growth factor, FVM cell: fetal ventral mesencephalon cell, MCAO: middle cerebral artery occlusion, MIAMI cell: marrow-isolated adult multilineage-inducible cell, MSC: bone marrow mesanchymal stem cell, NGC: nerve guidance channel, NGF: nerve growth factor, NSC: neural stem cell, PAM: pharmacologically active microcarriers, PC12: pheochromocytoma cell, PD: Parkinson’s disease, PDLLA: poly(d,l-lactic acid), PGA: poly(glycolic acid), PLGA: poly(lactic-*co*-glycolic acid), PLLA: poly(l-lactic acid), PNS: peripheral nervous system, rho^−/−^ mouse: rhodopsin knockout mouse, RPC: retinal progenitor cell, SC: Schwann cell, SD rats: Sprague–Dawley rats, SCI: spinal cord injury, SFI: sciatic nerve index, VEGF: vascular endothelial growth factor, 6-OHDA: 6-hydroxydopamine.

### 4.3. Natural Biomaterials

#### 4.3.1. ECM Components and Their Derivatives

##### Collagen

Collagen is one of the most extensively used components of ECM for neuroregeneration. Collagen has excellent cell-adhesion properties. This allows long-time survival and proliferation including angiogenesis of cells [132]. Spontaneous contraction of collagen gel occurs in the presence of cells. This is due to forces generated from cytoskeletal assembly and cell movements initiated by the formation of stable integrin-mediated attachments between collagen fibrils and cells [40]. Gelation of collagen can be induced by pH change, whereas its degradation is mediated by *in vivo* enzymatic mechanisms. Cell encapsulation within collagen matrix can be achieved under mild conditions. This enhances the survival of entrapped cells. Mechanical and degradation properties of collagen can be modulated by crosslinking [133] and the concentration of collagen used [21]. However, potential immune response may result if cross-species transplantation takes place or impurities exist. Currently, collagen-based nerve conduits/cuffs/wraps with FDA and/or CE approval include NeuraGen^®^, NeuraWrap™, Neuroflex™, NeuroMatrix™ and NeuroMend™ that are composed of bovine collagen type I as well as RevolNerv^®^ that is manufactured from highly purified porcine collagen type I/III.

Collagen-based cell-delivery systems have been studied as NGCs, fillers of NGCs and cell-encapsulating devices, coatings, scaffolds, *in situ* gelation platforms and for potential PNS and CNS treatments (Table 3A) [22,24,39,40,48,76,87,88,89,90,91,93,100,101,102,117,132,134,135,136,137]. Collagen offers a permissive environment to support cell survival, attachment, proliferation and differentiation. Collagen culture systems supported NSCs and NPCs to differentiation into functional neuronal circuits *in vitro* [138]. Also, culturing umbilical cord blood cells in a 3-D collagen system enhanced the expressions of a series of neurotrophic factors [139]. Lu *et al.* studied the reparative effects of implanting a collagen type I scaffold populated with MSCs into the lesion cavity of rats with TBI [134,135]. MSC neuroregenerative functions were enhanced when combined with collagen scaffolds. In comparison with control groups with scaffolds or cells only, MSC/collagen systems resulted in reduced lesion volume, enhanced angiogenesis and transcallosal neural fiber length as well as better sensorimotor and cognitive functions. Collagen scaffolds enabled the migration of transplanted cells to the lesion boundary zone while maintaining them in proximity to the injured tissue [134].

*In situ* gelling is another possible method to deliver collagen culture systems with minimal invasiveness. At constant pH, collagen becomes more viscous with increasing temperature. With the addition of different ECM proteins, laminin/collagen type I and fibronectin/collagen type I gels had different gelling temperatures that were physiologically relevant [136]. Although initial NSC survival was poor regardless of the delivery conditions, collagen-based gels sustained the survival of the remaining cells and improved cognitive functions in a mouse model of TBI. Since these collagen gels were able to fill irregularly shaped injury cavities and offered structural support, the delivered cells had a wider distribution than direct cell injections.

Some studies have pointed out that growth cone migration and neuroregeneration can be impeded by the presence of collagen due to a lack of matrix alignments or concentration-dependent abrogation [117]. Failure to recreate the aligned architecture of native tissues can lead to scarring and poor restoration of mechanical properties [140]. Therefore, many studies have focused on developing aligned anisotropic scaffolds. Keilhoff *et al.* studied the neuroregenerative effects of SC-seeded collagen type I/III scaffolds with different configurations in a relatively large 20 mm sciatic nerve defect in rats [24,132]. For cell-seeded collagen tubes, unlike the hollow configuration, no neuroregeneration was observed in tubes with a rather irregularly oriented inner skeleton structure [132]. Revascularizations were faster in seeded tubes with a hollow structure than those with an inner skeleton [24]. Also, neuroregeneration in seeded tubes with smaller hollow lumens was better than those with larger ones [132]. This was probably due to closer contacts between SCs and the regenerating fibers. One concern raised by the study was that animals with collagen implants showed a tendency for automutilating their treated digits [132]. This maybe contributed by mechanical irritations resulting from graft rigidity and the relatively large graft volume used. In order to mimic the spatial and molecular phenotype of autograft, Philips *et al.* developed a tethered and self-aligned collagen type I guidance conduit to deliver SCs to the PNS [40]. Uniaxial strain generated during the spontaneous gelling process in the presence of fibroblasts and SCs aligned the scaffold. SCs were found aligning along the directed matrix. Implanting this system into rats with 5 mm sciatic nerve defect showed greater axonal regrowth than controls. To fabricate a more compact and tissue-like scaffold with self-aligned SCs within a tethered collagen I matrix, interstitial fluid was removed by plastic compression [39]. These implants supported axonal regeneration across a 15 mm rat sciatic nerve gap after 8 weeks of implantation.

In short, collagen culture systems have great potentials in offering a generally biocompatible and ECM-mimicking environment for regenerating the nervous system. However, careful design and further *in vivo* investigations are needed to ensure treatment efficacy and biocompatibility of these potential cell-based therapies.

##### Gelatin

Gelatin is derived from denatured collagen and has been widely explored as macroencapsulating scaffolds and microcarriers for cell delivery to the CNS (Table 3B). The mechanical and chemical properties of gelatin can be modulated by covalently incorporating bioactive cues. Gelatin can also be mechanically stabilized by cross-linking to prevent its rapid clearance from the implanted site [3,12].

Gelatin has good biocompatibility and is often applied without immunosuppressant. An RPE cell sheet embedded into a gelatin sheet was transplanted into pigs without immunosuppression [141]. The gelatin-based implant was well tolerated with no observable infiltration of inflammatory cells. Transplanted cells were able to survive in the eye for up to 3 months and achieved some morphological rescue. However, sheet folding and degeneration of the inner layers of transplanted RPE cells were detected in some regions after prolonged implantation [141]. In another study, modified SCs with NT-3 production were co-cultured with differentiating NSCs modified to secrete tyrosine protein kinase C, a preferential binding receptor of NT-3, in a gelatin sponge and implanted into a rat SCI model [142]. The transplanted NSC-derived neural network integrated into the host network and formed a relay to conduct signals. Improvements in voluntary movement, body weight support, foot placement accuracy and coordination recovery were reported.

On top of macroencapsulating scaffolds, gelatin microcarrier systems have been evaluated in preclinical and clinical studies for CNS applications. Cross-linked porcine gelatin microspheres, with L-dopa-producing human RPE cells attached to their surfaces, were applied as a potential PD treatment under the name Spheramine^®^ (Bayer Schering Pharma AG, Berlin, Germany) in these studies. In animal models of PD, the microspheres allowed prolonged survival and monolayer distribution of the attached cells in the absence of immunosuppression. Unattached RPE cells were not able to survive well or to produce lasting therapeutic effect in the brain [143]. Long-term functional improvements as well as survival of RPE cells under no immunosuppressant were also observed in rat and non-human primate models of PD [143,144,145,146]. However, there were indications of chronic inflammation in rats at 5 months [146] as well as mild astroglial cell proliferation and inflammation, but no evidence of granulomatous or immune-mediated reactions in non-human primates [143]. Positron emission tomography imaging 2 months after transplantation in non-human primates revealed enhanced dopamine levels [145]. Phase I clinical trial on 6 patients with advanced PD showed that the device was well tolerated after 2 years of implantation [143]. Improvements in Unified Parkinson Disease Rating Scale motor disability score was detected 12 months after implantation and was sustained for 48 months, except for one patient who refused to be off the PD medications overnight. This was followed by a 2-year Phase II trial that recruited 71 patients. However, it was halted due to adverse effects reported [147]. Brain autopsy findings from a subject the treatment reviewed a drastic drop in overall survival of transplanted RPE cells after 6 months of implantation. Also, local inflammatory and astrocytic reactive changes were observed within the needle tract lesions and often within the microcarrier matrix material.

##### Laminin and Fibronectin

Laminin is the main non-collagenous component of the basal lamina that can promote neurite outgrowth and guide neurite development [9,12]. Fibronectin is a glycoprotein found in blood that is involved in wound healing and cell-adhesion as well as in axonal development and repair [21]. Laminin and fibronectin interact with cells and allow cell attachment to ECM as well as further signal transduction [17]. They support cell proliferation, migration and differentiation. Fragments of laminin and fibronectin, for example, peptides IKVAV, Tyr-Ile-Gly-Ser-Arg (YIGSR) and Arg-Gly-Asp-Ser-Pro (RGDSP) have been isolated and used for functionalizing scaffolds [148,149]. Studies have shown that laminin or fibronectin-coated scaffolds, even without cells, have more superior neural regeneration effects than control systems (reviewed in [17]). As a result, they are often employed for functionalizing NGCs, microcarriers, cell encapsulating devices and scaffolds to promote cell attachment, survival and motility (Table 3C,D) [41,59,62,64,80,120,127,128,136,150,151].

Laminin and fibronectin are often incorporated into hydrogels. Fibronectin- and laminin- incorporated collagen gels were explored as *in situ* gelling cell-carriers for TBI [136,150]. The gels were introduced with minimal invasiveness and were able to fit to the irregularly shaped injury cavity to provide physical support in a rat model of TBI. Although the delivery conditions used in this study did not protect NSCs from the initial decline in survival, NSC survival was enhanced after 8 weeks of transplantation when compared with direct injection of NSCs [136]. This study also reported that laminin-based scaffolds functioned better than fibronectin-based scaffolds in enhancing cell survival and functional recovery. In another study, the effects of fibronectin incorporation into alginate gel have been investigated. When fibronectin and SCs were added to the lumen of a biodegradable NGC filled with alginate hydrogel, these two factors showed additive effects on neuroregeneration across a 10 mm rat sciatic nerve gap [151]. A more significant rate of neuroregeneration and prolonged transplanted cell viability were observed after 6 weeks of implantation when compared with control conduits. Fibronectin improved the proliferation of transplanted SCs and host SCs that entered the NGC from the nerve stumps.

##### Fibrin

Fibrin is a key component in blood clotting and consists of a dense meshwork of cross-linked fibrinogen and thrombin. Its properties can be tailored by covalent modification. As with other naturally occurring biopolymers in the body, fibrin promotes cell adhesion with reduced immune response. It is explored as a filling medium for nerve conduits (Table 3E) [118,152].

In one study, fibrin glue with SCs or differentiated MSCs was incorporated into a biodegradable NGC and implanted across a 10 mm rat sciatic nerve defect [152]. The fibrin matrix was almost dissolved after 2 weeks with significantly better cell adherence to the NGC and cell distribution than those seeded in growth medium alone. A longer nerve regeneration distance was also observed in seeded fibrin conduits. Also, with daily injections of cyclosporine A, a fibrin NGC with fibrin filler was able to support the survival of MSCs, enhanced axonal regeneration and reduced immediate inflammation response when implanted across a rat sciatic nerve gap of 10 mm after 3 weeks of implantation [153].

##### Matrigel^®^

Matrigel^®^ contains a number of ECM molecules and growth factors and has been used as filler for nerve guidance channels of biodegradable [137,154,155] or non-biodegradable source [66,78] and cell-encapsulating device [92] (Table 3F). Although it had superior properties in promoting neurite growth and enhancing cell survival, proliferation and functioning, Matrigel^®^ derivation from a sarcoma cell line source has greatly limited its potential clinical use [11].

**Table 3:** Examples of biodegradable cell-carriers composed of Extracellular matrix components and their derivatives in preclinical animal studies and clinical trials.

Biomaterial	Form of Scaffold	Cell Type & Modified Factor Expression (if any)			Animal & Neurological Disorder Model	Outcomes			Ref.
**(A) Collagen**	Scaffold in cylindrical form				CNS				
	Collagen I	MSC			Rat TBI model (controlled cortical impact induced)	Reduced lesion volume, supported transplanted cell migration into lesion boundary zone, enhanced angiogenesis and improved sensorimotor cognitive functions.			[134,135]
	Scaffold formed by *in situ* gelation				CNS				
	Collagen I with laminin or fibronectin	NSC			Mouse TBI model (controlled cortical impact induced)	Scaffold conformed to injury cavity and supported cell migration into adjacent tissue. Improved cognitive function.			[136]
	Scaffold NGC				PNS				
	Collagen I/III (hollow or with collagen inner skeleton)	SC			Rat sciatic nerve defect (20 mm)	Neuroregeneration and revascularization were better in cell-seeded hollow collagen tubes, especially ones with reduced lumen. The inner skeleton impaired nerve regeneration independent of whether SCs were added or not.			[24,132]
	Collagen I (Tethered and uniaxially aligned)	SC & fibroblast			Rat sciatic nerve defect (5 mm)	SC aligned along the directed matrix. Improved axonal regrowth.			[40]
	Collagen I (Tethered and uniaxially aligned, followed by plastic compression)	SC			Rat sciatic nerve defect (5 mm and 15 mm)	SC aligned along the directed matrix. Improved neuroregeneration across the gap.			[39]
	With Matrigel^®^ and daily injections of FK506	SC			Mouse sciatic nerve defect (6 mm)	Improved axonal regeneration as well as functional recovery index in the hindpaw.			[137]
**(A) Collagen**	Scaffold				CNS				
	Cross-linked collagen I (with a small amount of collagen III) with longitudinal pore orientation	NSC			Rat SCI model (5 mm full-resection)			Defects had fewer cystic cavities and were filled largely with fibrous scars favorably aligned with the long axis of the spinal cord. Yet, no myelinated axon at the defect-center and recovery of bladder and hindlimb function were observed at the end of study.	[48]
	Filler of tubular NGC				PNS				
	Of silicone	NPC			Rat sciatic nerve defect (15 mm)			Improved axonal regeneration with detectable action potentials. Part of the transplanted cells differentiated into SC-like supportive cells.	[76]
	Of PLLA	SC			Rat sciatic nerve defect (12 mm)			Limited axonal regeneration and SFI recovery.	[117]
	Filler of cell-encapsulating device with hollow-fiber based membrane				CNS				
	Of P(AN/VC)	Fibroblast-NGF			i. Rat HD model (QA-induced) ii. Non-human primate AD model (fornix transection or aspiration)			Protected neurons from induced damages. Reduced rotational behavior in rodents.	[87,90,91]
		Fibroblast-CNTF			Rat and Non-human primate HD model (QA-induced)			Reduced the extent of induced striatal damage. Exerted trophic influence on degenerating striatal neurons as well as on critical non-striatal regions in non-human primates. Improved rotational behavior but not skilled forelimb function in rodents.	[88,89]
**(A) Collagen**	Of P(AN/VC)	PC12			Rat PD model (6-OHDA induced)		Continued presence of intrastriatal implants was required to maintain the reduction in rotation behavior. Device output was affected by the site of implantation.		[93]
	Of PES	Myoblast-CNTF			Rat ALS model (facial nerve axotomy)		Protected motor neurons from induced cell death.		[100]
		Fibroblast-CNTF			i. Phase I clinical trial: ALS patients; ii. Phase I clinical trial: HD patients		Prolonged device implantation and CNTF delivery were well tolerated. There were a varying number of surviving cells, hence, CNTF levels.		[22,101,102]
**(B) Gelatin**	Scaffold				CNS				
	-	RPE cell sheet			Healthy pig		Improved axonal regeneration with no immunosuppression administered. Yet, sheet folding and degeneration of inner layers of the transplanted cells were detected at some areas.		[141]
		SC-neurotrophin-3 & NSC-TrkC Co-culture			Rat SCI model (transection)		Transplanted NSC-derived neural network integrated into host neural networks and formed a relay to conduct signals. Improved voluntary movement, body weight support, accuracy of foot placement and coordination recovery.		[142]
	Tubular NGC				PNS				
	Of gelatin/poly(ε-caprolactone)	Exfoliated deciduous tooth-derived stem cells			Rat sciatic nerve defect (10 mm)		Enhanced transplanted cell survival and promoted axonal regeneration. Improved SFI and sensory functional recovery.		[156]
	Filler of tubular NGC				PNS				
	Of silicone	MSC			Rat sciatic nerve defect (15 mm)		Improved axonal regeneration, reduced loss of gastrocnemius muscle weight. Enhanced walking behavior and SFI recovery.		[79]
**(B) Gelatin**	Microcarrier				CNS				
	Cross-linked gelatin Spheramine^®^ (Bayer Schering Pharma AG)	RPE cell			i. Rat and non-human primate PD models (6-OHDA induced); ii. Phase I & II clinical trial: PD patients		In animal models, PAM supported transplanted cell survival in the absence of immunosuppression with enhanced dopamine levels. Yet, there were signs of chronic inflammation and mild astroglial cell proliferation. In Phase I clinical trial, PAM were well tolerated after prolonged implantation and had improved Unified Parkinson Disease Rating Scale motor disability score. Yet, Phase II trial was halted due to adverse effects reported.		[143,144,145,146,147,157]
**(C) Laminin**	Scaffold formed by *in situ* gelation				CNS				
	With Collagen I	NSC			Mouse TBI model (controlled cortical impact induced)		Scaffold conformed to injury cavity, supported cell migration into adjacent tissue and improved cognitive function. Outcomes were more favorable than fibronectin-based scaffolds.		[136]
	Filler of tubular NGC				PNS				
	Of PLGA conduit with five channels	SC			Rat sciatic nerve defect (7 mm)		Improved axonal regeneration comparable to autograft in mid-conduit region with axonal fibers detectable in all channels.		[41]
	Of PLGA membrane and fiber matrix	SC			Rabbit sciatic nerve defect (20 mm)		Improved axonal regeneration at mid-graft comparable to autografts.		[120]
	Of silicone, with laminin-coated multi-walled chitosan insert	MSC			Rat sciatic nerve gap (10 mm)		Improved axonal regeneration, ameliorated loss of gastrocnemius muscle mass and enhanced SFI recovery.		[80]
**(C) Laminin**	Coating of scaffold			CNS					
	Of PLGA and PLLA blend		RPC	Mouse rho^−/−^ model		Supported transplanted cell survival, differentiation, migration and integration into host retina.			[64]
	Coating of PAM/microcarrier			CNS					
	With VEGF release and laminin coating		Immortalized NSC	Rat stroke model (MCAO induced)		Attracted host endothelial cells into scaffold, established neovasculature in some parts and merely interspersed with NSCs in other parts. Evidence of hypervascularization present.			[127]
**(D) Fibronectin**	Scaffold formed by *in situ* gelation			CNS					
	With Collagen I		NSC	Mouse TBI model (controlled cortical impact induced)		Scaffold conformed to injury cavity, supported cell migration into adjacent tissue and improved cognitive behavior. Outcomes were less favorable than laminin-based scaffolds.			[136,150]
	Filler of tubular NGC			PNS					
	Of PHB conduit, with fibronectin in alginate gel		SC	Rat sciatic nerve defect (10 mm)		Improved transplanted cell survival. Supported proliferation of transplanted SCs and host SCs. Augmented axonal regeneration.			[151]
	Coating of PAM/microcarrier			CNS					
	With polylysine and fibronectin-like synthetic molecules coated PLGA (37.5:25), and release of NGF		PC12	Rat PD model (6-OHDA induced)		Supported transplanted cell differentiation and reduced cell death and proliferation. Reduced rotational behavior.			[59]
**(D) Fibronectin**	With allylamine deposition and fibronectin coated PLGA (85:15)		NSC	Rat stroke model (MCAO induced)		PAM filled lesion cavity. Scaffold integrated efficiently within host tissue forming a primitive neural tissue.			[62]
	With fibronectin and polylysine coated PLGA (37.5:25)		MIAMI cell	Rat stroke model (global cerebral ischemia induced)		Protected neurons in Cornu Ammonis layer 1 hippocampal region from ischemic death.			[128]
**(E) Fibrin**	Filler of tubular NGC			PNS/CNS					
	Of PDLLA		SC-D15A	Rat SCI Model (transection)		Increased vascularization but limited axonal regeneration response into and across the scaffold as well as hindlimb function recovery.			[118]
	Of PHB		SC& differentiated MSC	Rat sciatic nerve defect (10 mm)		Improved nerve regeneration distance.			[152]
**(F) Matrigel^®^**	Filler of tubular NGC			PNS/CNS					
	Of TMC-CL		SC	Rat SCI model (Partial transection)		Improved axonal regeneration.			[155]
	Of collagen, with daily injections of FK506		SC	Mouse sciatic nerve defect (6 mm)		Improved axonal regeneration as well as functional recovery index in the hindpaw.			[137]
	Of chitosan		SC	Rat sciatic nerve defect (12 mm)		Improved axonal regeneration, mid-shank circumference and nerve conduction velocity.			[154]
	Of silicone		SC & SC-FGF-2 isoforms	Rat sciatic nerve defect (10 mm)		Improved axonal regeneration, sensory and motion functional improvements. Different isoforms of FGF-2 exerted varying effects on the regenerating axons.			[78]
	Of P(AN/VC) and release of chABC		SC & OEG	Rat SCI Model (transection)		Improved axonal regeneration and enhanced coupling of forelimb and hindlimb.			[66]
**(F) Matrigel^®^**	Filler of cell-encapsulating device with hollow-fiber based membrane			CNS					
	Of P(AN/VC)		Fibroblast-NGF	Rat AD model (fimbria and dorsal fornix aspiration)		Ameliorated loss of septal choline acetyltransferase expression.			[92]

Abbreviations: AD: Alzheimer’s disease, ALS: Amyotrophic lateral sclerosis, chABC: chondroitinase ABC, CNS: central nervous system, CNTF: ciliary neurotrophic factor, FGF-2: fibroblast growth factor-2, HD: Huntington’s disease, MCAO: middle cerebral artery occlusion, MIAMI cell: marrow-isolated adult multilineage-inducible cell, MSC: bone marrow mesenchymal stem cell, NGC: nerve guidance channel, NGF: nerve growth factor, NPC: neural progenitor cell, NSC: neural stem cell, OEG: olfactory ensheathing glia, PAM: pharmacologically active microcarriers, P(AN/VC): poly(acrylonitrile-*co*-vinyl chloride), PC12: pheochromocytoma cell, PD: Parkinson’s disease, PDLLA: Poly (d,l-lactic acid), PES: poly(ethersulphone), PHB: poly(hydroxybutyrate), PLGA: poly(lactic-*co*-glycolic acid), PLLA: poly(l-lactic acid), PNS: peripheral nervous system, QA: quinolinic acid, rho^−/−^ mouse: rhodopsin knockout mouse, RPC: retinal progenitor cell, RPE cell: retinal pigment epithelial cell, SC: Schwann cell, SCI: spinal cord injury, SFI: sciatic function index, TBI: traumatic brain injury, TMC-CL: trimethylene carbonate-caprolacton block copolymer, 6-OHDA: 6-hydroxydopamine.

#### 4.3.2. Biodegradable Polymers of Non-Mammalian Origins

##### Alginate

Alginate is a biocompatible and naturally occurring polysaccharide commonly purified from brown algae. Purity of alginate is important in preventing undesirable host responses [14]. Gelation of alginate is triggered by divalent cations under physiologically relevant conditions. Degradation of alginate scaffolds *in vivo* is mainly due to the gradual exchange of gelling divalent cations with sodium ions. Alginate is often used as a cell encapsulation system and filler for biodegradable or non-biodegradable NGCs. It can form a semipermeable matrix that allows the transfer of therapeutic agents, gases, nutrients, and waste products between the immunoisolated cells and the host environment. Some targeted neurological conditions include AD [158], HD [159,160,161], PD [162], stroke or TBI [163,164,165], glioma [166], chronic pain [94,167] and sensory diseases [70,168] (Table 4A).

Alginate microcapsules have been used to provide immunoisolated cell delivery to the CNS and are well tolerated in animal models in a range of neurological conditions. In one study, alginate microcapsules with endostatin-expressing modified fibroblasts were implanted into a rat glioma model [166]. Viable transplanted cells, maintained factor secretion and reduced tumor volume were detected in treated rats after 4 months of implantation. These rats survived longer than untreated rats. In another study, alginate microcapsules were able to support the survival of genetically modified MSCs that expressed neuroprotective glucagon-like peptide-1 and exert neuroprotective effects on TBI rats after 2 weeks of implantation [163]. Garcia *et al.* further demonstrated the mechanical stability of these carriers in a mouse AD model [158]. Alginate beads with modified CNTF-secreting myoblasts were found intact after 8 months of implantation and resulted in significant improvements in cognitive functions.

In order to strengthen the mechanical properties of alginate scaffolds, some studies have included semipermeable polylysine or polyornithine coating in the alginate microcapsule design for CNS application. CP cells from a range of donors, including xenogeneic sources, have been encapsulated in these coated carriers and applied into various animal models of HD, stroke and sensory loss with generally good biocompatibility. One study showed that the process of serial coating and encapsulation did not impact cell survival [161]. Minimal gliotic reactions after 3 days of implantation were observed in rats receiving encapsulated CP cells when compared with the control group with unencapsulated ones [164]. Also, a separate study demonstrated that encapsulated CP cells remained metabolically active for at least 6 months post-implantation [70]. Coated-microcarriers with CP cells reduced striatal infarct volume of rats with stroke and resulted in motor and neurological improvements [164,165]. Similarly, these implants were able to reduce the lesion volume of rats [159] and non-human primate [161] displaying HD symptoms. Improved motor functions were detected in these rats. In the case of auditory neuron rescue in deafened cats, Wise *et al.* demonstrated that neuroprotective effects were most prominent when encapsulated cells were applied in combination with chronic electrical stimulation [70]. Other studies have applied coated carriers with chromaffin cells or PC12 cells to rodent models of PD and chronic pain [162,167]. They were well tolerated *in vivo* and remained intact although some were deformed probably as a result of the mechanical forces exerted by the host brain. Improvements in behavioral tests after prolonged implantation were detected. In another study involving the encapsulation of BDNF-overexpressing SCs showed enhanced auditory neuron survival in deafened guinea pigs [168].

As alginate is biologically inert, some studies have functionalized alginate with ECM components to further enhance its potential as a cell transplantation matrix. An enhanced rate of axonal regeneration was detected when liquid fibronectin was added to the alginate matrix of a biodegradable NGC that was seeded with SCs [151].

Apart from biodegradable cell-encapsulating devices, alginate has been applied to immunoisolated cells in devices with semipermeable non-biodegradable outer membrane. A P(AN/VC) encapsulating alginate system with chromaffin cells have been applied to patients with chronic pain in a Phase I trial without immunosuppressant. Retrieved devices showed sustained cell survival and no fibrous coating on device despite moderate therapeutic outcomes after prolonged implantation [23].

##### Agarose

Similar to alginate, agarose is a linear polysaccharide obtained from seaweed and must undergo extensive purification to prevent immune responses after implantation [21]. Reversible gelation of agarose is temperature-triggered. Agarose has been explored as a cell encapsulation system in preclinical studies (Table 4B). BDNF-overexpressing fibroblasts in agarose were used to coat the electrodes of a cochlear implant [169]. No noticeable inflammation was found surrounding the agarose matrix despite signs of cell migration away from the matrix in some implants. Although cell survival decreased over the course of implantation, the system was able to demonstrate neuroprotection on spiral ganglion neurons in the deafened guinea pigs after 48 days of implantation.

##### Chitosan

Chitosan is a derivative of chitin, which is widely found in crustacean shells, fungal cell walls and insect exoskeletons. Chitosan has similar molecular structure to glycosaminoglycan, allowing its interaction with ECM molecules. Gelation and degradation of chitosan are mediated by ionic or chemical cross-linking with glutaraldehyde and enzymatic hydrolysis respectively [19]. As chitosan lacks bioactivity and that pure chitosan cannot support adequate cell adhesion, it is often used in combination with other materials for nerve tissue engineering applications (Table 4C) [19,114]. Chitosan-silk fibrin wraps with adipose-derived stem cells or SCs were able to improve neurological and functional recovery in rats with 10 mm partial sciatic nerve defect after 24 weeks of implantation [170]. In other studies, cell-carrying chitosan NGCs with different biodegradable fillers, including collagen, Matrigel^®^ and PLGA, and different cell types, such as SCs, MSCs and NSCs, have been applied to animal models of peripheral nerve defects [42,122,123,154,171]. Generally, these conduits were able to encourage axonal regeneration. In one study, a chitosan NGC with Matrigel^®^ and SCs of autologous or MSC-derived sources was implanted across a 12 mm rat sciatic nerve defect [154]. Similar axonal regeneration, recovery in mid-shank circumference of hindlimbs and nerve conduction velocities were observed for both types of SCs and were better than PBS-filled control conduits after 3 months of implantation. Chitosan conduits with physical cues incorporated have been shown to enhance neuroregeneration [42,122,123]. Conduits with aligned PLGA inserts were able to promote better axonal regeneration and functional recovery than scaffold alone when implanted in dogs with a large sciatic nerve gap of 60 mm [122] and non-human primates with a median nerve gap of 50 mm [123] after 12 months of implantation.

##### Dextran

Dextran is a bacterial-derived polysaccharide and has been applied for coating neural implants due to its anti-thrombotic properties as well as resistance to cell and protein adhesion [21]. In order to encourage cell-material interactions for tissue engineering purposes, dextran has been chemically modified for better cell adhesion or fabricated with porous structures to encourage tissue infiltration [172].

A collagen-coated dextran microcarrier (Cytodex^®^ 3, Sigma-Aldrich, St. Louis, MI, USA) was the first microcarrier explored for conveying cells into the brain, reported by Cherksey *et al.* (Table 4D) [173]. When chromaffin-cell attached Cytodex^®^ 3 microcarriers were introduced into rat PD models, these microcarriers were able to support prolonged cell survival at the transplanted site and resulted in more significant and longer-lasting behavioral improvements than direct cell injections over 8 months [173] and 12 months [174] of study. Enhanced survival of microcarrier attached human fetal ventral mesencephalic neurons after 3 months of implantation in PD rats without immunosuppressant was also demonstrated in another study [175]. These microcarriers were generally well tolerated with no inflammation when implanted in the striatum [173] despite one study, which reported a low level, yet ongoing glial response after 14 months of implantation [174]. However, such host response may be beneficial to protecting the long-term survival of transplanted cells. Also, similar functional rescuing results could be obtained from chromaffin cells attached to dextran-based microcarriers or glass bead microcarriers [173]. This suggests that transplanted cell survival was more related to the provision of attachment by the microcarriers than biomaterial used.

##### Poly(hydroxybutyrate) (PHB)

PHB is produced naturally by certain species of bacteria and can be degraded by the same mechanics as the synthetic polyesters mentioned in Section 4.2.1 [4]. PHB has been applied as a cell-carrying NGC (Table 4E). PHB conduits with SCs were able to promote neuroregeneration in a 10 mm sciatic nerve gap in rats [176]. When alginate, fibronectin and SCs were added to a PHB conduit, these conduit-filling components presented an additive effect on axonal regeneration as well as survival and proliferation SCs [151]. However, the application of PHB is hindered by concerns over contamination from bacteria components [3].

This section presented common biomaterial candidates for temporary to long-term cell and growth factor delivery. Although most of combinatory therapies with cells and biomaterials are still in preclinical development, they offered promising opportunities for repair and regenerating the nervous system.

**Table 4:** Examples of non-animal derived natural biomaterials as cell-carriers applied in animal or clinical trials.

Biomaterial	Form of Scaffold	Cells & Modified Factor Expression (if any)	Animal & Neurological Disorder Model	Outcomes	Ref.	
**(A) Alginate**	Microcapsule		CNS			
	-	Fibroblast-endostatin	Rat glioma model	Supported transplanted cell survival, sustained factor delivery and reduced tumor volume. Animals survived longer.	[166]	
		Myoblast-CNTF	Mouse AD model (amyloid-β oligomer-induced)	Supported transplanted cell survival and improved cognitive function.	[158]	
		MSC-GLP-1	Rat TBI model (controlled cortical impact)	Supported transplanted cell survival and exerted neuroprotective effects.	[163]	
	With polylysine or polyornithine coating	CP cell	i. Rat stroke model (MCAO induced) ii. Rat and non-human primate HD model (QA induced) iv. Cat deaf model (aminoglycoside induced)	Supported transplanted cell survival. Reduced striatal infarct or lesion volume and improved in motor and neurological functions. Auditory neuron rescue most prominent when combined with chronic electrical stimulation.	[70,159,161,164,165]	
		Chromaffin cell	Rat PD model (6-OHDA induced)	Supported transplanted cell survival and reduced rotational behavior.	[162]	
		PC12	Rat chronic pain model (Chronic constriction induced)	Supported transplanted cell survival and suppressed cold allodynic behavior.	[167]	
		SC-BDNF	Guinea Pig deaf model (ototoxin induced)	Enhanced auditory neuron survival.	[168]	
	Filler of Tubular NGC		PNS			
	Of PHB conduit, with fibronectin in alginate gel	SC	Rat sciatic nerve gap (10 mm)	Improved transplanted cell survival. Supported proliferation of transplanted SCs and host SCs. .Augmented axonal regeneration.	[151]	
**(A) Alginate**	Filler of cell-encapsulating device		CNS			
	Of P(AN/VC)	Chromaffin cell	Phase I clinical trial: Chronic pain patients	Devices were retrievable. Supported transplanted cell survival without immunosuppressant. Yet, only moderate therapeutic outcomes, including morphine intake and pain ratings improvements, were detected in some patients.	[23]	
**(B) Agarose**	Coating of electrode		CNS			
	-	Fibroblast-BDNF	Guinea pig deaf model (kanamycin and ethacrynic acid induced)	Despite decreasing cell survival over implantation period, device exerted neuroprotection on spiral ganglion neurons.	[169]	
**(C) Chitosan**	Tubular NGC		PNS			
	With collagen and NGF	NSC	Rabbit facial nerve defect (10 mm)	Increased axonal regeneration.	[171]	
	With Matrigel^®^	SC	Rat sciatic nerve defect (12 mm)	Improved axonal regeneration, nerve conduction velocity and mid-shank circumference.	[154]	
	Of chitosan conduit, with PLGA fiber inserts	MSC	Canine sciatic nerve defect (60 mm)	Improved axonal regeneration and functional recovery.	[122]	
		MSC	Non-human primate median nerve defect (50 mm)	Improved axonal regeneration and functional recovery.	[123]	
	With multi-channeled conduits	MSC	Rat sciatic nerve defect (8 mm)	Improved axonal regeneration. SFI recovery comparable to autograft.	[42]	
**(C) Chitosan**	Scaffold		PNS			
	Of chitosan-silk fibroin	Adipose-derived stem cell or SC	Rat partial sciatic nerve defect (10 mm)	Improved axonal regeneration, gastrocnemius muscle mass and SFI recovery.	[170]	
	Of silicone, with laminin-coated multi-walled chitosan insert	MSC	Rat sciatic nerve gap (10 mm)	Improved axonal regeneration, ameliorated loss of gastrocnemius muscle mass and enhanced SFI recovery.	[80]	
**(D) Dextran**	Microcarrier		CNS			
	With collagen coating Cytodex^®^ 3 (Sigma-Aldrich)	Chromaffin cell	Rat PD model (6-OHDA induced)	Supported transplanted cell survival, reduced lesion volume and sustained improvements in rotational behavior and motor functions	[173,174]	
**(E) PHB**	Tubular NGC		PNS			
	-	SC	Rat sciatic nerve gap (10 mm)	Improved axonal regeneration.	[176]	
	With fibronectin in alginate gel matrix	SC	Rat sciatic nerve gap (10 mm)	Improved transplanted cell survival. Supported proliferation of transplanted SCs and host SCs. Augmented axonal regeneration.	[151]	
	With fibrin	SC & differentiated MSC	Rat sciatic nerve defect (10 mm)	Improved nerve regeneration distance.	[152]	

Abbreviations: AD: Alzheimer’s disease, BDNF: brain-derived neurotrophic factor, CNS: central nervous systems, CNTF: ciliary neurotrophic factor, CP cell: choroid plexus cell, GLP-1: Glucagon-like peptide-1, HD: Huntingdon’s disease, MCAO: middle cerebral artery occlusion, MSC: bone marrow mesenchymal stem cell, NGC: nerve guidance channel, NGF: nerve growth factor, NSC: neural stem cell, P(AN/VC) poly(acrylonitrile-*co-*vinyl chloride), PC12: pheochromocytoma cell, PD: Parkinson’s disease, PHB: poly(hydroxybutyrate), PLGA: poly(lactic-*co*-glycolic acid), PNS: peripheral nervous system, QA: quinolinic acid, SC: Schwann cell, SFI: sciatic function index, TBI: traumatic brain injury, 6-OHDA: 6-hydroxydopamine.

## 5. Conclusions and Future Perspectives

To date, full functional regeneration of the human nervous system remains a challenge, especially for injuries of the CNS and critical sized gaps in the PNS. Increasing knowledge in the characteristics and properties of different cell and biomaterial candidates as well as their interactions with the host microenvironment takes us one step closer to achieving this goal. In this review, we have focused on the most common non-biodegradable and biodegradable scaffolding materials for conveying cells to targeted sites for prolonged neuroactive factor delivery as well as cell replacement. Non-biodegradable cell-encapsulating systems have been shown to be well tolerated *in vivo* and retrievable after prolonged implantation in clinical trials. These systems are promising in providing a continuous drug delivery at the site of injury or tissue loss. However, these materials have limited ability in promoting neuroregeneration. As for biodegradable materials, due to their tunable mechanical, biochemical and degradation properties, they can be applied as long-term cell encapsulation drug delivery or culturing device, and also temporary scaffolds for protecting transplanted cells and mechanically supporting injured tissue. However, synthetic biodegradable materials do not have the inherited bioactivity as in natural materials, whereas for natural materials, reaction and degradation rates are often more difficult to control when compared with synthetic materials. To maximize the merits of different types of scaffolding materials, hybrid scaffolds and scaffold functionalization with physical, biochemical and electrical cues can be applied. These modifications allow researchers to have a more precise control over cell-material interactions and to manipulate the spatial and temporal events during neuroregeneration more effectively. The combined strategies can potentially target multiple aspects of the neurological disorder.

Although many innovative and promising cell-conveying scaffolds have been developed, some issues have to be addressed before they can be applied in clinical settings. As these scaffolds are meant for extended applications, more data is needed regarding the long-term performance of the implant system. This includes safety, sustainability and controllability of factor release, well-regulated cell survival, growth, differentiation, migration and tissue re-organization, as well as therapeutic efficacy and long-term interactions with other living components in animal and clinical studies. Additional factors to consider are tunable properties for individualized applications in addition to consistency and ease of scaling up in manufacturing process.

Better understanding of injury progression mechanisms in terms of anatomical and physiological changes on top of the effectiveness of different neuroactive drug and cell candidates is essential for developing the next generation of cell-carrying scaffolds. This allows researchers to tailor conveyer systems that encourage more dynamic interactions with the transplanted cells and host tissue at various stages of regeneration. Multiple-component and multiphasic scaffolds can be developed to provide a more precise spatial and temporal control over cell behavior and function, such as differentiation and migration, and tissue restructuring. With the combination of different biomaterials, cells, instructive physical, biochemical and electrical cues, therapeutic potential of cell-based polymer scaffolds can be further amplified. As these systems involve more components, more complex analysis will be required to fully characterize them.

In conclusion, the integration of biomaterial scaffolds and cell-based therapies have great potentials in promoting functional repair and structural regeneration in the human nervous system.
